# Exploring the Acceptability and Feasibility of a Self-directed Approach to Identifying Health Priorities in a Sample of Southern Older African American Adults with Multiple Chronic Conditions

**DOI:** 10.1007/s40615-025-02469-8

**Published:** 2025-05-23

**Authors:** Deborah Ejem, Marie Bakitas, Raegan W. Durant, Tamara Nix Parker, Kwaku Duah Oppong, Jessica Esterson, J. Nicholas Odom, Rachel D. Wells, Kenneth Boockvar, Mary E. Tinetti

**Affiliations:** 1 School of Nursing, University of Alabama at Birmingham, Birmingham, AL USA; 2 Center for Palliative and Supportive Care, Heersink School of Medicine, University of Alabama at Birmingham, Birmingham, AL USA; 3 Division of General Internal Medicine and Population Science, Heersink School of Medicine, University of Alabama at Birmingham, Birmingham, AL USA; 4 School of Nursing, The University of Texas at Austin, Austin, TX USA; 5 Division of Gerontology, Geriatrics, and Palliative Care, Heersink School of Medicine, University of Alabama at Birmingham, Birmingham, AL USA; 6 Department of Internal Medicine, Section of Geriatrics, School of Medicine, Yale University, New Haven, CT USA

**Keywords:** African Americans, Multiple chronic conditions, Family caregivers, Culturally tailored interventions, Patient-clinician communication, Telehealth feasibility

## Abstract

**Objectives:**

To evaluate the cultural acceptability and feasibility of the self-directed “My Health Priorities” (MHP) web-based program in older Southern African Americans (AAs) with multiple chronic conditions (MCCs) and their family caregivers (FCGs).

**Design:**

A multi-method formative evaluation study (NIH Stage 1a) to explore patients’ and FCGs’ experiences with the MHP web-based program, a component of the patient priorities care approach. Interviews were analyzed using the constant comparative method and thematic analysis. Participants rated usability via the system usability scale (SUS) (scores range from 0 to 100). Sample characteristics were analyzed using SAS and SPSS.

**Setting:**

A primary care clinic in a southeastern U.S. academic medical center.

**Participants:**

Fifteen older AAs with MCCs (≥ 65 years old, diagnosed with ≥ 2 chronic conditions) and their adult AA FCGs (≥ 18 years old).

**Results:**

Participants generally found the program acceptable but difficult to navigate on devices other than computers. Suggestions included redesigning the avatar for cultural relevance, optimizing functionality for mobile devices, and offering strategies to address challenging patient-clinician interactions. Patients rated usability at 75.31 ± 14.63 (good usability), while FCGs rated it at 30.13 ± 4.31 (indicating limited usability). Study measures required 30 min to complete, while the intervention took 60 min. Participants completed 81% of study measures.

**Conclusions:**

Established web-based programs may be acceptable to AA patients and their caregivers, but specific technical and content aspects may need to be revised to make the programs more suitable for AAs. Design refinements should account for the differing usability experiences reported by AA patients and their caregivers.

Trial Registration: NCT05129709.

## Introduction

Over two-thirds of older (≥ 65 years old) African American (AA) adults in the United States (US)—many living in the South—have multiple chronic conditions that require ongoing healthcare management, such as heart disease [1, 2], cancer [3, 4], diabetes [5], and lung disease [6]. Managing these conditions often requires clear and effective communication between patients and their clinicians; yet, older AA adults with multiple chronic conditions (MCCs) frequently face challenges in articulating their values and preferences [7]. Such communication is essential for delivering patient-centered care, optimizing treatment outcomes, and ensuring medical care aligns with patients’ values and priorities [8–10]. However, AAs are rarely asked about their values and preferences during clinical encounters [11, 12]. Many do not initiate communication to convey this information, often due to feelings of disempowerment and mistrust [11–13]. Structural disparities, socio-economic challenges, and historical inequities can collectively contribute to diminished agency, hindering individuals from taking an active role in healthcare interactions [8, 14, 15]. Moreover, fear of judgment, bias, or inadequate care can also impede establishing collaborative healthcare relationships [15–17]. Challenges in articulating healthcare preferences and goals increase the risk of receiving care that is not aligned with individual values, potentially leading to adverse outcomes such as poorer quality of life, heightened symptom burden, and increased psychological distress [18]. Consequently, older AAs with MCCs may further mistrust and disengage from the healthcare system, potentially delaying treatment of MCCs [11]. Delayed treatments for serious illness result in higher rates of healthcare utilization, costs, poor end-of-life outcomes, and mortality among older AAs with MCCs compared to their white counterparts [19–25].

Appropriate care for people with MCCs should be aligned with patients’ specific health goals and preferences. Patient-centered care, defined by the National Academy of Medicine as “care that is respectful of, and responsive to, individual patient preferences, needs, and values and ensuring that patient values guide all clinical decisions” [26] should also be culturally responsive. Culturally responsive care considers cultural and social factors in managing clinical encounters with patients from diverse backgrounds [27–29]. While several culturally responsive health promotion interventions exist [29–36], there is a lack of culturally responsive patient-centered care models effectively activating AAs with MCCs with complex medical care needs to engage in discussions with their clinicians about their healthcare values and preferences.

The self-directed “My Health Priorities” Identification Program (“My Health Priorities” Program), developed by Tinetti and colleagues, is part of the patient priority care (PPC) approach. The PPC is an evidence-based values solicitation model that reduced patient-reported treatment burden and unwanted diagnostic tests [37]. “My Health Priorities” is a web-based program that includes four online interactive modules designed to help patients identify specific and actionable health outcome goals and healthcare preferences, resulting in a printable communication guide tailored to address the patients’ specific health concerns to empower patients’ conversations with their clinicians during clinic visits. However, because the PPC has only been tested in a racially homogenous sample of Northeastern older White adults [38–40], it remains unclear whether the “My Health Priorities” Program would be equally acceptable in a population of Southern older AAs with MCCs. Consistent with the NIH Stage Model 1a [41], we conducted a formative evaluation of the current “My Health Priorities” Program to determine cultural acceptability and feasibility among a sample of older Southern AAs with MCCs in a primary care setting.

## Methods

### Study Design, Sample, and Setting

Patient and family caregiver (FCG) dyads participated in this formative evaluation study, known as the Black Health Identification Project (B-HIP), from March 2022 to January 2024. This study used a multi-methods approach to explore their experiences with the “My Health Priorities” Program. Both qualitative and quantitative data were collected in parallel but analyzed separately, with no formal integration of findings—making this a multi-method, rather than a mixed-methods, design. Older Southern AA patients with MCCs, receiving primary care at the University of Alabama at Birmingham (UAB), a single tertiary academic medical center in the southeastern United States, along with their FCGs, were purposively sampled. Patient inclusion criteria included (1) identifying as AA; (2) being 65 years or older; (3) having MCC, defined as at least two of the following chronic conditions: cancer, heart disease, kidney disease, liver disease, chronic obstructive pulmonary disease, asthma, osteoarthritis, and rheumatoid arthritis per medical record; (4) English-speaking; (5) without dementia or cognitive impairments, per medical record, affecting decision-making; (6) having reliable internet or telephone access via computer, tablet, or smartphone; and (7) having a FCG willing to participate in the study. Patients were excluded if they had evidence of (1) electronic medical recorder (EMR)-documented axis I psychiatric disorder (schizophrenia, bipolar disorder), or active substance use disorder, or (2) if they resided in a nursing home or assisted living facility.

Consistent with other studies [20, 42–44], FCG was defined as “a relative, friend, or partner with whom you have a close relationship and who assists you with your medical care and who may or may not live in the same residence and who is not paid for their help.” The FCG did not have to be a blood or adoptive relative or household member to serve as an FCG. Inclusion criteria for FCGs were (1) identifying as AA; (2) being at least 18 years old; (3) identified by the patient as his or her primary FCG; (4) English-speaking; and (5) having reliable internet or telephone access via computer, tablet, or smartphone.

This study was approved by the UAB Institutional Review Board (IRB#3000007623).

### Recruitment Strategy

Based on prior successful recruitment procedures [45], patients from four participating primary care clinics at the UAB Kirklin Clinic were identified through an EMR review using inclusion criteria #1–5 (listed above). Potentially, eligible AA patients were sent an opt-out letter and a study flyer outlining the study details. The materials informed them that they would be contacted in 2 weeks unless they opted out by calling the study phone number. After the 2-week period, study staff contacted patients who had not opted out. If the patient agreed to participate and identified an FCG, the staff member requested the FCG’s contact information to provide study details. Interested patients and FCG completed the IRB-approved verbal informed consent process. Patients and FCGs were compensated $25 for each data collection activity they participated in (baseline questionnaire, follow-up questionnaire, and semi-structured interviews), with a maximum total of $75 for completing all study activities.

### Study Procedures

Patients and FCGs completed baseline questionnaires separately by phone with the study’s Clinical Research Coordinator (CRC). Following completion, the CRC scheduled a convenient time for both the patient, with the assistance of the FCG, to complete the “My Health Priorities” Program on their computer, smartphone, or tablet, with the CRC joining them over Zoom or by phone. Patients and FCGs went through each of the four modules on their own, with the CRC standing by to answer any technical questions. Immediately after completing the “My Health Priorities Program,” the CRC conducted semi-structured acceptability interviews (see Box) with the patient and FCG dyad jointly. Twelve weeks after completing baseline questionnaires, patients and FCGs complete follow-up questionnaires separately by phone with the study’s CRC.

### Qualitative Acceptability Interviews

Digitally recorded acceptability interviews lasted between 30 and 45 min. The interview guide included 17 open-ended questions exploring patients and FCGs’ perspectives regarding (1) how well the program addressed patients’ values, as well as cultural and spiritual concerns; (2) the potential impact of the program on communication about health concerns with clinicians and family members; (3) opinions on caregiver involvement in the program; and (4) general feedback on the appearance and functionality of the “My Health Priorities” Program (see Box 1 for Abbreviated Interview Guide).

**Box 1** Abbreviated acceptability interview guide.

**Table:** 

Patients and Family Caregivers
1) What would you change about the web-based Self-directed “My Health Priorities” Identification Program? a. What do you think about how the program looks? b. What are your thoughts on Dave? 2) How well did the program address your specific values? 3) How well did the program address your cultural concerns? 4) How well did the program address your spiritual concerns as it relates to your/your loved one’s health? 5) How has the program influenced how you think about the treatments you are taking for your conditions? 6) How has the program influenced your ability to speak with your family members about your health concerns? 7) Some people have said that this program makes them feel more empowered to speak with their providers about what is important to them. What do you feel about that statement? **Family Caregivers Only** 8) What do you think about your inclusion in the program?

Developed by a multidisciplinary team of co-investigators (D.E., M.B., J.N.O., R.W.D., J.E., M.T.), the semi-structured acceptability interview guide drew on expertise in geriatrics, palliative care, cancer, heart failure, medical sociology, and qualitative research methods. The semi-structured acceptability interview guide was pilot tested with 5 AA patient-FCGs dyads to ensure question clarity before the study commenced. The interview guide was revised once during the study based on the ongoing performance of interview questions.

### Participant-reported Outcomes

#### Program Usability Measures

The primary outcome measure of the formative evaluation was study feasibility. After engaging with the “My Health Priorities” Program, patients and FCGs were asked to complete the10-item system usability scale (SUS) [46] to assess its ease of use and participant satisfaction with the program. Scores range from 0 to 100, with high scores indicating better usability. We also collected the time to complete the program, and the device used (i.e., computer, smartphone) to access the program.

#### Outcome Measures for Future Efficacy Intervention

To assess outcomes in a future intervention study, we tested the acceptability, feasibility, and timing of collecting quantitative measures related to illness and FCG experiences at baseline and 12 weeks post-baseline. Patient participants were asked to complete: (1) the Older Patient Assessment of Chronic Illness Care (O-PACIC) [47], a 10-item assessment of older patients’ perceptions of primary care delivery (score range, 10–50; higher score indicates more positive perceptions); (2) the Treatment Burden Questionnaire (TBQ) [48], a 13-item scale measuring patients’ perceptions of quality of life and treatment burden related to chronic illness (score range, 0–130; higher scores indicates higher perception of treatment burden); and (3) the CollaboRATE scale [49], a 3-item tool used to measure patients’ perceptions of how much they feel involved in shared decision making with their clinicians (scores range from 1 to 9, with higher score indicating better shared decision making). (4) Shared care instrument (SCI) [50], a 19-item measure, which measured their perception of communication exchange between the patient and FCG regarding the illness experience (score range, 10–50; higher scores represent greater degree of perceived shared care).

FCGs were asked to complete the 10-item Bakas caregiver outcomes (BCOS) [51] which measures FCG social function, subjective well-being, and somatic health (scores range, 15 to 105; higher scores indicate positive caregiver outcomes) and SCI (described above).

Demographic measures collected from patients and FCGs included sex, age, education, marital status, religious affiliation, diagnoses, and the patient completed item stating their relationship to theFCG.

### Data Analysis

#### Qualitative Data Analysis

Verbatim transcripts of patients’ and FCGs’ interviews were entered into NVivo [47]. Analysis commenced when qualitative interviews began, with transcripts examined for themes related to the cultural appropriateness of “My Health Priorities” Program; additional emerging codes across cases were operationally defined and entered into a formal codebook. Three researchers (D.E., T.N.P., K.D.O.) independently coded the transcripts and met to compare coding and to discuss any discrepancies or areas of ambiguity. Transcripts were then analyzed within and across participants using an iterative constant comparative method [48] and content thematic analysis [49]. To ensure the trustworthiness of the data, an audit trail was also maintained. A priori categories and newly emerging themes were collapsed into three main domains: (1) Acceptability of the “My Health Priorities” Program; (2) Accessibility of the “My Health Priorities” Program; and (3) Suggested changes to the “My Health Priorities” Program.

#### Quantitative Data Analysis

Analyses were performed using SAS 9.4 and IBM SPSS Statistics Version 28. Descriptive statistics were used to summarize participants’ demographic characteristics and reported outcomes. Feasibility was assessed by evaluating completion rates and adherence to the data collection schedule. As the primary aim of this study was to assess the feasibility of collecting the selected instruments, no statistical comparisons or tests of association were conducted.

## Results

**Fig. 1:**
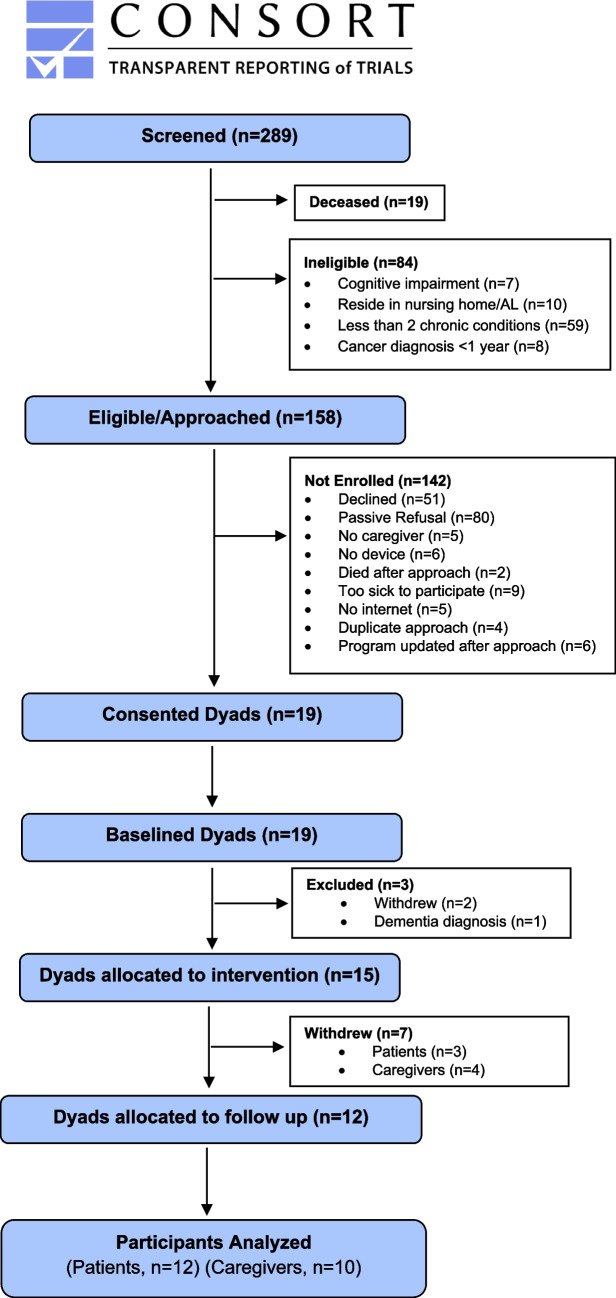
Consort diagram

From March 2021 to February 2024, 15 patient-FCG dyads participated in this formative evaluation of the “My Health Priorities” Program. Initially, 289 patients’ charts were reviewed to assess eligibility. Study personnel subsequently contacted 159 patients to invite them to participate in the program, which included a one-time semi-structured acceptability interview and pre-and post-intervention surveys. A total of 19 patients and 19 caregivers enrolled in the study, with 15 dyads receiving the intervention. Of these, 12 patients and ten caregivers completed all study activities (Fig. 1).

### Sample Characteristics

Sample characteristics of enrolled patients and caregivers are summarized in Table 1. Patients were an average age of 72.8 years, mostly female (68%), married (58%), and retired (68%). Most patients endorsed a Christian faith (84%), regularly attended religious services (58%), and had recently prayed for their own health (74%). Most patients had not been hospitalized in the past year (84.2%), and 31.6% had either completed an advance directive or designated a healthcare power of attorney. The most common diagnosis among patients was type 2 diabetes (47.4%), lung disease (47.4%), heart disease (47.4%), and osteoarthritis (47.4%).

**Table 1:** Patient characteristics and outcomes scores overview

		Enrolled (*n* = 19)		Complete (*n* = 12)		Incomplete (*n* = 7)		Effect size
Patient characteristics		*n*	%	*n*	%	*n*	%	Cramer's *V*
Age in years (mean [SD])		18	72.8 [8.5]	12	71.1 [6.8]	6	76.2 [11]	Cohen’s *d* = 0.61
Gender								0.19
	Male	6	31.6	3	25	3	42.9	
	Female	13	68.4	9	75	4	57.1	
Marital status								0.52
	Never married	1	5.3	0	0	1	14.3	
	Married	11	57.9	7	58.3	4	57.1	
	Divorced	3	15.8	1	8.3	2	28.6	
	Widowed	4	21.1	4	33.3	0	0	
Religion								0.33
	Protestant	2	10.5	2	16.7	0	0	
	Other Christian	16	84.2	9	75	7	100	
	Other	1	5.3	1	8.3	0	0	
Attend religious services								0.29
	Never	1	5.3	1	8.3	0	0	
	Occasionally	6	31.6	3	25	3	42.9	
	Regularly	11	57.9	7	58.3	4	57.1	
	Not applicable	1	5.3	1	8.3	0	0	
Ever prayed for own health								0
	Yes	19	100	12	100	7	100	
In past month, prayed for own health								0.04
	No	5	26.3	3	25	2	28.6	
	Yes	14	73.7	9	75	5	71.4	
Other members in household								
	Spouse	10	52.6	7	58.3	3	42.9	0.15
	Parents	1	5.3	0	0	1	14.3	0.31
	Children	5	26.3	3	25	2	28.6	0.04
	Friends	0	0	0	0	0	0	0
	Significant other	0	0	0	0	0	0	0
	Other relatives	5	26.3	4	33.3	1	14.3	0.21
	Live alone	2	10.5	1	8.3	1	14.3	0.09
Employment status								0.55
	Full-time	1	5.3	1	8.3	0	0	
	Part-time	1	5.3	0	0	1	14.3	
	Retired	13	68.4	8	66.7	5	71.4	
	Student	1	5.3	0	0	1	14.3	
	Unemployed and looking for work	1	5.3	1	8.3	0	0	
	Unemployed due to disability/illness	2	10.5	2	16.7	0	0	
Type of medical insurance								0.55
	Private/commercial	9	47.4	5	41.7	4	57.1	
	Affordable Care Act Medicare	1	5.3	1	8.3	0	0	
	Medicaid	2	10.5	0	0	2	28.6	
	Medicare and private don't know	7	36.8	6	50	1	14.3	
Diagnosis								
	Type 2 diabetes	9	47.4	9	75	0	0	0.72
	Lung disease	9	47.4	6	50	3	42.9	0.07
	Heart disease	9	47.4	5	41.7	4	57.1	0.15
	Osteoarthritis	9	47.4	4	33.3	5	71.4	0.37
	Chronic kidney disease (any stage)	8	42.1	4	33.3	4	57.1	0.23
	Cancer	6	31.6	5	41.7	1	14.3	0.28
	Rheumatoid arthritis	2	10.5	2	16.7	0	0	0.26
	HIV	2	10.5	0	0	2	28.6	0.45
	Osteoporosis	2	10.5	2	16.7	0	0	0.26
	Chronic liver disease or cirrhosis	1	5.3	0	0	1	14.3	0.31
	Type 1 diabetes	1	5.3	1	8.3	0	0	0.18
	Lupus	1	5.3	1	8.3	0	0	0.18
In past 2 months, number of times admitted to hospital								0.33
	0	16	84.2	11	91.7	5	71.4	
	1	2	10.5	1	8.3	1	14.3	
	2	1	5.3	0	0	1	14.3	
In past 2 months, involved with palliative care program								0.31
	No	15	88.2	8	80	7	100	
In past 2 months, seen by provider								0.24
	No, only a palliative care provider	2	11.8	2	16.7	0	0	
	Yes	12	70.6	8	66.7	4	80	
	No Response	3	17.6	2	16.7	1	20	
Completed advanced directive, power of attorney								0.28
	No	13	68.4	7	58.3	6	85.7	
	Yes	6	31.6	5	41.7	1	14.3	
Have do-not-resuscitate order (DNR)								0.28
	No	13	68.4	7	58.3	6	85.7	
	Yes	6	31.6	5	41.7	1	14.3	
		Mean	SD	Mean	SD	Mean	SD	Cohen’s *d*
System usability scale (SUS)* (*n* = 16)		75.31	14.63	NA	NA	NA	NA	NA
Older patients assessment of chronic illness care (O-PACIC) mean score		30.47	7.57	26.97	4.7	36.48	8.04	1.56
Treatment burden questionnaire (TBQ) global score		34.22	31.99	29.52	26.92	42.29	40.26	0.40
CollarboRATE		6.98	1.08	7.00	0.93	6.92	1.62	0.08
	Understanding mean score	6.91	1.18	6.88	1.17	7.00	1.41	0.10
	Listening mean score	7.13	1.27	7.25	1.00	6.75	2.06	0.39
	Effort mean score	6.91	1.08	6.88	0.91	7.00	1.63	0.11
Shared care instrument (SCI)								
	Patient communication	10.59	5.76	10.19	6.95	11.29	3.15	0.19
	Patient decision-making	26.87	2.69	27.13	2.26	26.43	3.46	0.25
	Patient reciprocity	32.84	3.75	32.67	4.12	33.14	3.29	0.12
*SUS pre only								
**SUS [1(strongly disagree)—5 (strongly agree)]								
Notes:								
Cohen’s *d*: small = 0.2, medium = 0.5, large = 0.8, computed with pooled SD								
Cramer’s *V*: small = 0.1, medium = 0.3, large = 0.5								

FCGs were an average age of 52.6 years, mostly female (85%), married (60%), and 47% were employed full time. Like patients, most FCGs identified as Christian (74%), though a smaller proportion (48%) reported regularly attending religious services. However, 84% of FCGs had recently prayed for their own health. In terms of relationships, patients were most commonly the parent or grandparent of the FCG (53%), followed by the spouse (26.7%), and then a sibling or friend (20%) Table 2.

**Table 2:** FCG characteristics and outcomes scores overview

		Enrolled (*n* = 19)		Complete (*n* = 10)		Incomplete (*n* = 9)		Effect size
Caregiver characteristics		*n*	%	*n*	%	*n*	%	Cramer’s *V*
Age in years (mean [SD])		19	53.3 [13.4]	10	55 [11.6]	9	51.3 [15.7]	Cohen’s *d* = 0.27
Gender								0.15
	Male	5	26.3	2	20	3	33.3	
	Female	14	73.7	8	80	6	66.7	
Marital status								0.27
	Never married	6	31.6	2	20	4	44.4	
	Married	10	52.6	6	60	4	44.4	
	Divorced	3	15.8	2	20	1	11.1	
Religion								0.37
	Catholic	2	10.5	2	20	0	0	
	Other Christian	14	73.7	6	60	8	88.9	
	Other	3	15.8	2	20	1	11.1	
Attend religious services								0.33
	Never	1	5.3	0	0	1	11.1	
	Occasionally	9	47.4	4	40	5	55.6	
	Regularly	9	47.4	6	60	3	33.3	
Ever prayed for own health								0
	Yes	19	100	10	100	9	100	
In past month, prayed for own health								0.12
	No	3	15.8	2	20	1	11.1	
	Yes	16	84.2	8	80	8	88.9	
Other members in household								
	Spouse	10	52.6	6	60	4	44.4	0.16
	Parents	4	21.1	0	0	4	44.4	0.54
	Children	4	21.1	3	30	1	11.1	0.23
	Friends	0	0	0	0	0	0	0
	Significant other	1	5.3	1	10	0	0	0.22
	Other relatives	4	21.1	2	20	2	22.2	0.03
	Live alone	0	0	0	0	0	0	0
Employment status								0.51
	Full-time	9	47.4	5	50	4	44.4	
	Part-time	2	10.5	1	10	1	11.1	
	Retired	5	26.3	4	40	1	11.1	
	Student	1	5.3	0	0	1	11.1	
	Unemployed and looking for work	1	5.3	0	0	1	11.1	
	Unemployed due to disability/illness	1	5.3	0	0	1	11.1	
Common diagnosis								
	Heart disease	9	47.4	5	50	4	44.4	0.06
	Cancer	6	31.6	4	40	2	22.2	0.19
	Lung Disease	9	47.4	5	50	4	44.4	0.06
	Chronic kidney disease (any stage)	8	42.1	4	40	4	44.4	0.04
	Chronic liver disease or cirrhosis	1	5.3	0	0	1	11.1	0.25
	Type 1 diabetes	1	5.3	0	0	1	11.1	0.25
	Type 2 diabetes	9	47.4	9	90	0	0	0.9
	Lupus	1	5.3	1	10	0	0	0.22
	Osteoarthritis	9	47.4	3	30	6	66.7	0.37
	Rheumatoid arthritis	2	10.5	2	20	0	0	0.33
	Sickle cell disease	0	0	0	0	0	0	NA
	Multiple sclerosis	0	0	0	0	0	0	NA
	Parkinson’s disease	0	0	0	0	0	0	NA
	HIV	2	10.5	0	0	2	22.2	0.36
	Osteoporosis	2	10.5	2	20	0	0	0.33
Completed advanced directive, power of attorney								0.12
	No	16	84.2	8	80	8	88.9	
	Yes	3	15.8	2	20	1	11.1	
Have do-not-resuscitate order (DNR)								0.25
	No	18	94.7	10	100	8	88.9	
	Yes	1	5.3	0	0	1	11.1	
		**Mean**	**SD**	**Mean**	**SD**	**Mean**	**SD**	**Cohen’s *d***
Bakas caregiver outcomes (BCOS) total score		68.05	14.24	73.09	9.07	62.44	17.23	0.79
System usability scale (SUS)* (*n* = 16)		30.13	4.31	NA	NA	NA	NA	NA
Shared care instrument (SCI)								
	Caregiver Communication	12.81	4.23	13.25	4.95	12.25	3.37	0.23
	Caregiver Decision Making	22.17	5.95	23	5.57	21.13	6.62	0.31
	Caregiver Reciprocity	29.63	7.14	31.64	4.91	27.13	8.94	0.65
*SUS pre only								
**SUS [1(strongly disagree)—5 (strongly agree)]								
Notes:								
Cohen’s *d*: small = 0.2, medium = 0.5, large = 0.8, computed with pooled SD								
Cramer’s *V:* small = 0.1, medium = 0.3, large = 0.5								

### Qualitative Results

#### Feasibility and Acceptability Outcomes

Patients and FCGs qualitative interviews lasted an average of 40 min (range, 30–45 min) and centered around three domains: (1) Acceptability of the “My Health Priorities” Program; (2) Accessibility of the “My Health Priorities” Program; and (3) Suggested changes to the “My Health Priorities” Program (Table 3).

**Table 3:** Domains, themes, and supporting quotes from participants

Domain	Theme	Exemplar quote
1. Acceptability of the “My Heath Priorities” Program	a. Program helped patients to be better advocates for themselves	*“Well, because a lotta things that [program] asked was very helpful. When you go to the doctor, you can more ask more questions [instead of] just sittin’ there and listenin’ to them.”* (Patient 124, 66 y/o)
	b. Program helped FCGs get on the same page with patients	*“We could see the same things, know what her priorities are. I think that it should happen, instead of caregivers guessing or whatever…I think, again, it’s helpful to really put’em on the same page.”* (FCG 255, 42 y/o female)
	c. Program is not culturally specific	*“To me, when I think of culture, I think about from my perspective as a[n] African American woman. The questions that they ask is general. You know what I’m sayin’? It wasn’t’pecific things like for a[n] African American woman we don’t get the treatment that we deserve. You know what I’m sayin’? We don’t have access—a lot of us don’t have access to mammograms, Pap smears, things that’s gonna help us. I didn’t see anything that really jived with me. To me, it’s more general.’Cause anybody can say, ok, you need to go to the doctor. You need to do this. To me, for African Americans, it need to be a little bit tweaked. That’s all I’m sayin’.”* (FCG 141, 60 y/o female)
	d. Program does not address spiritual needs, but reactions were mixed on whether it was needed	*“I only saw maybe one or two questions on spiritual concerns. That's okay with me for the simple reason that you can get into a lot of different kinda crazy stuff if you have too many questions. Mm-hmm.”* (Patient 215, 68 y/o female) *“I don't think they asked enough specific questions because in African American community. Religion plays a very significant part as far as health. Going back to years and years.”* (FCG 005, 44 y/o female)
2. Accessibility of the “My Health Priorities” Program	a. Program is not optimized for use on cellphone or tablets	*Interviewer:“What did you like the least about the My Health Priorities program?”* *Interviewee:“Not being able to see my screen like I supposed to [laughter].”* (Patient 206, 68 y/o female)
	b. Program made some feel more empowered to use technology while intimidating others	*“Okay. Well, I feel like using this technology. It's really inspired me even more… Because I love the technology that I'm facin'even today.”* (Patient 005, 87 y/o female) “*The computer is kinda off limits to me. Other than that, [the “myHP” Program” was] okay. I just need to be on the computer a little bit more that what I normally do. Other than that, it was very helpful.”* (Patient 124, 66 y/o male)
3. Suggested Changes to the “My Health Priorities” Program	a. Program’s avatar should be changed to a more representative character	*“Dave was okay, but he not really representative of [all] people, but he's a representative of some more active, I guess, people, but not than other people that they can't get around.”* (Patient 111, 74 y/o female)
	b. Program should incorporate health information relevant to African Americans with MCCs and their FCGs	*“There’s a lotta people who have depression and anxiety, but I didn’t see anything about that on that. You know what I’m sayin’? “ (*FCG 141, 60 y/o female)
	c. Program should provide guidance on how to approach patient-clinician relationships that may be challenging or unproductive	*“Yeah,’cause as African American, you feel like you have to stay with that doctor till you die. That’s not the case, but I think as a culture, we find a doctor and stick with’em regardless if they good or bad.”* (Patient 283, 68 y/o female)

#### Domain: Acceptability of the “My Health Priorities” Program

Most participants found the content of the “My Health Priorities” Program acceptable for documenting key health values and priorities. Participants felt it could help them advocate more effectively for their healthcare needs, while caregivers appreciated how it clarified patients’ health priorities. However, many participants noted that the program lacked focus on issues, e.g., diabetes and high blood pressure management, important to AAs with comorbidities and did not sufficiently address participants’ spiritual needs.

##### Theme: The program helped patients to be Better Advocates for Themselves

Most study participants thought that the “My Health Priorities” Program was useful in helping patients to be more active in healthcare discussions with clinicians and to be better advocates for care that aligned with their healthcare priorities. For example, one participant shared, *“I think it’s useful because it really gets you a chance to express yourself. This we don’t get a lot of*” (Patient 005). Another participant described how the program helped her to set realistic health goals, saying, *“This one would have been my favorite [program], and talking about the goals I need, what’s realistic, what’s not realistic. Just to be happy”* (Patient 241). Other participants also valued the program’s potential to facilitate future conversations with providers, explaining, *“[The program will] help me in the long run because I look at’em. Why I do some[thing] then I put it back [out when] I talk to the healthcare peoples about”* (Patient 141).

##### Theme: The Program Helped FCGs Get on the Same Page with Patients

Many FCGs stated that they liked the program because they felt it helped them to “get on the same page” as patients. One FCG noted,

*“I liked when [the program was] askin’ about her, what’s most important to her … because I think we (the caregiver) could get caught up in what we think is most important to us, but that really doesn’t matter if it is not important to them (the patient)…If anything does happen, we all know what this person[‘s] wishes are”* (FCG 126).

Another FCG described how they appreciated the program because it “*informed [them] on what’s going on with [the patient]”* and how they could be of help (FCG 215).

##### Theme: Participants Desired the Program to be More Culturally Relevant

Despite positive feedback, some participants thought that the “My Health Priorities” Program did not adequately address cultural issues relevant to AAs. *“To me, when I think of culture, I think about from my perspective as a[n] African-American woman” says one FCG, “The questions that they ask is general…It wasn’t’pecific things like for a African-American woman we don’t get the treatment that we deserve”* (FCG 141). Similarly, a patient commented, *“I just think it was just more general. I could say it’s just more general. It’s wasn’t on either way. It was just more down the middle, I guess you could say it”* (Patient 111).

##### Theme: The Program Does Not Address Spiritual Needs, But Reactions were not Mixed on Whether it was Needed

Nearly all participants observed that the program did not address spiritual values, though opinions varied on whether it was needed. When asked whether spiritual concerns were covered appropriately, one participant said, *“I only saw maybe one or two questions on spiritual concerns. That’s okay with me for the simple reason that you can get into a lot of different kinda crazy stuff if you have too many questions”* (Patient 215). Similarly, a FCG felt that the program did not need to include spiritual aspects: *“To me, as is’cause some people might not like the spiritual parts of it, and you don’t wanna offend people”* (FCG 141). In contrast, another FCG thought the program needed more emphasis on spirituality, explaining, *“I don't think they asked enough specific questions because in African American community. Religion plays a very significant part as far as health. Going back to years and years”* (FCG 005).

#### Domain: Accessibility of the “My Health Priorities” Program

Participants accessed the program on various devices, including laptops, desktop computers, cellphones, and tablets. While many found the program easier to navigate on larger screens, smaller devices posed challenges with reading and engaging with the content. For example, when asked about what one cellphone user thought was her least favorite part of the program, she remarked,* “Not being able to see my screen like I supposed to [laughter]”* (Patient 206). Another commented that viewing the program on a device did not compare to reading something tangible: *“That was nothing like getting something in the mail. Putting it on your dinette table sittin’ and readin’ it. It was nothing like that. That made it a little bit harder to digest”* (Patient 077). Reactions to the use of technology varied: some felt intimidated by having to use a computer—*“The computer is kinda off limits to me”* (Patient 124)—while others appreciated the flexibility of completing the program at home. A few participants reported feeling more empowered to use technology after engaging with the program: “ *It really inspired me even more… Because I love the technology that I am facing even today”* (Patient 005).

#### Domain: Suggested Changes to the “My Health Priorities” Program

Participants offered suggestions to improve the content and delivery of the “My Health Priorities” Program—specifically modifying the program’s avatar to better reflect users, incorporating information about issues central to older AAs with MCCs, such as medication management, chronic disease management (i.e., diabetes), and mental health issues, such as depression and anxiety. Caregivers suggested that the program should also include space to discuss self-care. Finally, participants suggested that the program should include guidance on how to approach patient-clinician relationships that may be challenging or unproductive.

Theme: The Program’s Avatar Should be Changed to a More Representative Character.

Currently, the avatar named “Dave” guides users through the program and provides example responses in each module. While some participants found Dave’s responses helpful for decision-making, many expressed that he did not accurately represent them. Participants described Dave as racially ambiguous and able-bodied, stating that he did not resonate with their experiences. For example, one patient-care dyad remarked, “*We’re African American”* (FCG 005) *and “I wasn’t too hot on Dave”* (Patient 005). They specifically highlighted activities that Dave engaged in, noting*, “That was one of the things that I did not understand. Because one of the activities [Dave] mentioned was playing poker and having friends. I did not like that… Dave shouldn’t have been an example for me to try to go by”* (Patient 005). Another participant expressed a desire for different social activities, stating that rather than wanting to spend more time with friends like Dave, he/she would prefer *“to live with my family members more, goin’ out to eat with them”* (Patient 124). Additionally, another patient commented that Dave appeared healthier than many users of the program: *“Dave was okay, but he’s not really representative of [all] people. He represents some more active, I guess, people, but not others who can’t get around”* (Patient 111).

Theme: The Program Should Incorporate Health Information Relevant to Older AAs with MCCs and Their FCGs.

Participants suggested that the program include information specific to health issues important to AAs with MCCs. Some mentioned the need for the program to improve how it collects information on medications. Currently, participants are limited to entering a maximum of four medications, even though many are prescribed more than four. Several participants requested additional space to list their medications along with the purposes of each. As one participant suggested, *“Like my medication. Put down two meds, but not what the meds were for. Again, I think the program would be nice if that was more”* (Patient 077). Another participant suggested that there should also be space in the program for participants to record information on any health procedures that they have undergone, as well as an area to reflect on their experience with those procedures: *“What worked for you, what has worked for you, and what didn’t work for you during that time, you was getting the procedures done”* (Patient 111).

Other suggested changes included providing some content on chronic illnesses that disproportionately affect old AAs. One patient stated*, “Maybe ask us more questions that have to deal with us.’Cause like ask about your—’cause number one people with diabetes is African Americans. Go more in detail about things”* (Patient 141). A small number of participants also highlighted that mental health conditions should also be considered, as they are prevalent chronic conditions. *“They asked a lotta questions, but that’s a lotta people right now who’s really depressed because of the COVID things that’s goin’ on. I think they need to address that, depression and anxiety”* (FCG 141).

Finally, an FCG suggested that the program include information about self-care*. “I have a chronic condition. Maybe, it could help a caregiver too. That one exercise could help them to how they need to take care of themselves… because that’s an important part of caregiving. You don’t wanna wear yourself out”* (FCG 241).

Theme: Desire for More Guidance on How to Approach Patient–Clinician Relationships that may be Challenging or Unproductive.

Participants described experiences of “*feeling minimized” “ignored”* and “*misunderstood*” with some even reporting feelings of “*humiliation”* and being perceived as “*aggressive*” when having discussions with their clinicians. Many participants emphasized the need for the program to focus on how to approach health conversations with clinicians and how to manage difficult patient-clinician relationships. Participants expressed that older AAs are often not aware of their choices when it comes to healthcare*. “[I want] to make sure that people know that they don’t have to—that the doctor, patient relationship should be a partnership. It just shouldn’t be what they say and not consider your—what you think about it”* (Patient 241). Another participant expressed frustration with not being able to effectively communicate their symptoms with their clinicians.

*“I don't think they understand the pain and the position you be in. They probably do understand it, but I just feel like I need to have more of—let’s see, how I'm gonna put it, like there are more things than just medicine…I be trying to tell her what actually how I feel, but I just don’t feel I’m being understood sometimes”* (Patient 111).

Participant suggestions included preparing the participants to complete the program prior to interaction with clinicians so that they can prepare themselves for subsequent conversations with clinicians. In particular, a participant said, *“I felt maybe [the study team] should have sent an email [to me] with those the [“My Health Priorities” Program] responses on there”* (FCG 005)*.* Another FCG suggested that the program *“give [the patient] some insight on the things that she’s takin’ and what she’s takin’ them for and in terms of givin’ feedback, askin’ questions about somethin’ different if needed.”* (FCG 215). Similarly, another patient suggests that the program *“go more into [detail] encouraging people that they need to talk, actually talk to their doctor, ask questions. If they are not happy with it, seek some health care somewhere else”* (Patient 241).

Participants also suggested that the program include a follow-up component after patients have had conversations with providers to check in*. “Maybe, and I know this may not be a part of it, a follow-up to see where—how it—if the— if I had used what I got from it, if I, would I get—if it improved my relationship with my physician”* (Patient 241). A few participants thought the program should also include communication training for clinicians*. “I think it needs to be more questions in there about how [clinicians] will respond to the patient and their reaction to the patient. And not just look at it as a job”* (FCG 206).

### Quantitative Results

#### Completion Rates for Study Measures

Study measures at baseline, interview, and post-intervention required approximately 30 min each to complete (90 min total). The intervention itself took approximately 60 min. Of the 114 possible sets of quantitative measures (19 dyads × 2 persons per dyad × 3 time points), 93 sets (81%) were completed. Missing data accounted for 21 sets (19%), distributed as follows: 15 sets (13%) were missing due to failure to reach participants, 4 sets (4%) were missing because the patient was no longer eligible for the study (caregiver disclosed a dementia diagnosis documented at an external institution but not in the study institution’s EMR), and 2 sets (2%) were missing due to caregiver withdrawal from the study.

#### Program Engagement and Participant Health Priorities

On average, the “My Health Priorities” program took 87.9 min to complete. Of the fifteen patient-caregiver dyads, nine accessed the program using a desktop or laptop, while six used a mobile device such as a cellphone or tablet. When asked to identify the “One Thing” they most wanted to address with their health care providers, the majority of patients (*n* = 5) selected pain management. Other priority areas included managing symptoms of inflammatory conditions (*n* = 2), challenges with medical equipment and diagnostic procedures (*n* = 2), balance issues (*n* = 1), fatigue (*n* = 1), and constipation (*n* = 1).

#### System Usability Scale (SUS)

Sixteen patients and 16 FCGs responded to the SUS after engaging with the “My Health Priorities” Program. Patients rated the “My Health Priorities” Program with a 75.31 ± 14.63, indicating good usability. In contrast, FCGs rated the program with a score of 30.13 ± 4.31 reflecting the program’s limited usability among this group.

## Discussion

This formative evaluation study evaluated the cultural acceptability and feasibility of the “My Health Priorities” Program among older Southern AAs with MCCs in a primary care setting. It also assessed the ability of this patient and family population to complete pre- and post-intervention measures addressing patients’ experiences with care (O-PACIC), perceptions of treatment burden (TBQ), clinical shared decision-making (CollaboRATE), caregiver quality of life (BCOS), and patient-caregiver perceptions of care (SCI). Program acceptability interviews revealed that while patients and FCGs generally found the program acceptable, they highlighted areas for improvement, including a lack of cultural specificity and usability challenges on tablets and cellphones. Participants suggested redesigning the program’s avatar to reflect greater cultural relevance, incorporating health information tailored to older AAs with MCCs and their caregivers, and providing strategies to navigate challenging patient-clinician relationships. The quantitative usability measure showed good usability among patients and limited usability among FCGS. Participants reported that outcome measures were easy to complete. These results offer preliminary evidence supporting the acceptability and feasibility of the “My Health Priorities” Program, highlighting the need for further refinement to improve cultural relevance and usability.

The findings that the program lacked cultural specificity align with prior research that emphasizes the importance of tailored interventions for improving healthcare outcomes of AA MCCs populations. Studies have consistently identified the absence of cultural competencies in research studies as a significant barrier to recruitment, retention, and meaningful engagement with minority groups [52–54] Without culturally specific approaches, study staff may find it difficult to build trust and effectively connect with participants [55]. Additionally, clinical characteristics of older AA adults, such as lower physical functionality, a higher number of medications, and other epidemiological aspects of chronic disease highlight the importance of accurate representation in study materials. While these characteristics are not cultural, per se, they contribute to a nuanced understanding of this population and the need for authentic depictions in interventions. Culturally tailored interventions, including accurate representation, have been shown to increase patient disease-specific knowledge, satisfaction, and access to healthcare [56–59]. To increase the acceptability and feasibility of the “My Health Priorities” program, future iterations should incorporate tailored imagery, content, and language. These modifications could potentially lead to deeper connections with study participants, ultimately improving health outcomes among AAs with MCCs.

It is important to note that patients rated the usability of the program as good, while FCGs found it more difficult. Potentially, FCGs were more involved with facilitating navigation of the program, whereas patients primarily provided pertinent information. This dynamic may have influenced their ratings, as caregivers—who are typically more familiar with technology and more accustomed to navigating healthcare systems—might have had higher expectations for ease of use. Acceptability interviews revealed that many found the program difficult to use because it was not optimized for use on devices other than desktop or laptop computers. The difficulty in using the program on tablets and cellphones, along with FCGs lower usability ratings on the SUS, highlights persistent barriers to technology use that disproportionately affect the under-resourced and minority populations. Limited broadband access, inadequate devices, and low digital literacy are well-documented roadblocks to web-based technology adoption in rural and under-resourced areas, further compounding disparities in these areas [60, 61]. It is important that future versions of the “My Health Priorities” Program improve accessibility and usability by addressing device compatibility issues within this population.

Consistent with prior research, we found that AA patients with MCCs struggled with communicating with their clinicians [7, 11, 62, 63]. Perhaps, the program and qualitative interviews, which came before the post-intervention assessment, identified underlying issues with the patient-provider relationship. Participants expressed sentiments such as “*humiliation, feeling minimized, and ignored,*” “*being misunderstood,*” and feeling as though they were perceived as “*aggressive*” by their clinician. These sentiments align with existing literature that underscores the significant challenges that AAs with serious illness frequently encounter in clinical interactions, including experiences of bias and stigmatization [62, 64–66]. Patients who did not feel that their concerns have been adequately heard by their clinicians are more likely to express feelings of mistrust and low satisfaction, leading to decreased adherence to treatment plans and poorer health outcomes [14, 64, 67, 68].

There were mixed reactions regarding the need for additional content addressing patients’ spiritual needs. Research strongly supports integrating spiritual concerns into health discussions to provide holistic care, as neglecting these concerns can negatively impact physical and mental health, while addressing them can strengthen therapeutic relationships and help patients cope with illness [69–71]. This is particularly relevant for older AAs in the South with chronic illnesses, who often prioritize religion and spirituality [72, 73]. However, in this study, patients emphasized the importance of aligning the intervention with their health experiences rather than their spiritual beliefs. This suggests that, while patients may find spirituality important in their lives, they may view it as a separate domain from their medical care—one that should be addressed through other sources, such as faith communities or personal practices. Future adaptations of the intervention should allow for the flexible inclusion of spiritual content based on patient preference.

Cultural and implicit bias in clinical encounters may be contributing to the negative perceptions expressed by participants. Systemic inequities may also influence how AA patients with MCCs are treated and perceived in healthcare settings, further amplifying communication challenges. The study participants’ experiences emphasize the importance of cultural humility among clinicians, especially those who work with diverse populations. While the broader PPC Approach includes content on patient-clinician communication, future iterations of the “My Health Priorities” Program should consider incorporating modules that empower patients to advocate more effectively for themselves in healthcare encounters in the web-based program. Additionally, educational content should be developed to enhance clinicians’ training on cultural nuances of communication and implicit bias. Clinicians can support more active patient participation in healthcare conversations by using plain language, encouraging questions, incorporating patients’ preferences and values in decision-making, using culturally relevant material when available, and celebrating small patient successes. Addressing these systemic barriers will require healthcare systems to implement structural changes aimed at reducing inequities. Furthermore, healthcare institutions should focus on making systemic changes that will reduce health disparities and foster equitable care for all they serve.

This study has a few notable limitations. First, consistent with NIH stage model 1a [41] our sample size was designed to explore the acceptability “My Health Priorities” Program and the feasibility of participants’ completing study activities. As such, the sample size was relatively small and limited to AAs with MCCs. Caution should be taken not to generalize findings to other racial, ethnic, or geographic populations. Second, we did not collect education of patients and FCGs which could have provided further insight into the relationship between education, digital literacy, and health literacy. Finally, the study experienced a relatively low recruitment rate (12%, with 19 patients consented out of 158 approached). This can be attributed to several factors. Early in the study, staff turnover contributed to recruitment challenges. Additionally, participation was initially limited to individuals with access to a desktop or laptop computer, as the “My Health Priorities” Program was optimized for use on those devices. This requirement excluded potential participants; however, it was revised approximately 6 months into the recruitment phase to allow individuals with smartphones or tablets to participate. Despite those difficulties, qualitative saturation was achieved. Another barrier to recruitment was the requirement for participants to have an FCG. Furthermore, approximately 1 year into the study, significant modifications were made to the “My Health Priorities” Program. As a result, we were unable to complete the study with participants who were already in the recruitment pipeline, in order to avoid collecting data on experiences that may have varied significantly between those using the earlier and revised versions of the program.

## Conclusion

Older adults with complex healthcare needs require clear and effective communication between patients and their clinicians, particularly when soliciting patients’ values and preferences to guide care. However, older AA adults with MCCs often face challenges in articulating their values and preferences. As a result, they are more likely to experience fragmented, inefficient, and insufficient care. This fragmented care can lead to unnecessary patient burden and potential harm, particularly when treatments conflict, such as in the case of the adverse effects of polypharmacy. While disease-specific care guidelines may be appropriate for some older adults with few functional limitations, they do not adequately address the needs of those with medical complexities [74]. Minority and under-represented populations face additional challenges, including systemic inequities and communication barriers, which exacerbate health disparities. These findings underscore the critical importance of culturally tailored interventions to address healthcare disparities and improve patient- and caregiver-centered outcomes. Future telehealth research should place a strong emphasis on patient engagement in the design process, particularly to explore the specific communication barriers faced by AA patients with MCCs. Incorporating interactive skill-building components, such as role playing, may help patients more confidently communicate their healthcare priorities during clinical encounters. Additionally, further studies are needed to evaluate interventions that enhance shared decision-making, foster mutual understanding in clinical interactions, and accommodate varying levels of technological proficiency. Longitudinal studies will also be crucial to assess the program's long-term effectiveness, including its ability to sustain reductions in treatment burden and improve shared healthcare decision-making. By continuing to investigate these factors, we can create a more inclusive healthcare environment that fosters trust, improves outcomes, and promotes equity in care delivery.

## Data Availability

Data supporting the findings of this study are available upon reasonable request from the corresponding author.

## Code Availability

The code used for analyses in this study is available upon reasonable request from the corresponding author.
